# Dr. Baillie

**Published:** 1860-10

**Authors:** 


*Dr. Baillie* affirms that women are less subject to disease of the arterial system than men, owing to their more sedentary life, producing less impetus in the circulation, and less liability to diseased alterations of structure. B.

